# Immune Profiling the Axilla with Fine Needle Aspiration Is Feasible to Risk-Stratify Breast Cancer

**DOI:** 10.3390/cancers18020251

**Published:** 2026-01-14

**Authors:** Jasmine A. Gore, Amy M. Llewellyn, Chuen Y. R. Lam, Jacqueline D. Shields, Kalnisha Naidoo

**Affiliations:** 1 Translational Pathology, The Rayne Institute, Comprehensive Cancer Centre, King’s College London, London SE5 9NU, UK; 2 Department of Cellular Pathology, King’s College Hospital, London SE5 9RS, UK; ringo.lam@nhs.net; 3 Translational Medical Sciences, Centre for Cancer Sciences, Biodiscovery Institute, University of Nottingham, Nottingham NG7 2RD, UK

**Keywords:** axillary lymph node clearance, regional metastasis, fine needle aspiration, lymph node immunophenotyping, patient stratification

## Abstract

**Background**: Axillary lymph node (ALN) metastasis is a critical prognostic determinant in breast cancer (BC) that informs surgical management. However, surgically clearing the axilla carries morbidity, so less invasive methods of risk-stratifying patients are needed. ALN fine needle aspiration (FNA) is currently used to detect BC metastases, but these samples also contain immune cells. **Methods**: Cells obtained via FNA from BC-patient-derived ALNs were analysed using flow cytometry. **Results**: FNA acquires sufficient leukocytes for comprehensive immunophenotyping of reactive, patient-derived ALNs. All CD4^+^ and CD8^+^ T-cell subsets (naïve, terminal effector, central memory, and effector memory) and rarer (<2%) natural killer (NK) and plasmacytoid dendritic cell (pDC) populations are represented. Importantly, the immune-cell profile of one reactive ALN appears to reflect the immune status of the patient’s axilla. Furthermore, FNA captures immune differences between patients with ≤1 or ≥2 metastatic ALNs. Increased numbers of naïve CD4^+^ T cells, but fewer terminal effector, central memory, and effector memory subpopulations, were obtained from patients with ≥2 metastatic ALNs. Moreover, despite their sparse distribution pattern on whole-section immunohistochemistry (WSI), FNA revealed that CD56^+^ NK cell activation receptors were decreased in patients with ≥2 metastatic ALNs. Finally, FNA captured a decrease in pDCs in patients with ≤1 metastatic ALNs, despite their clustered distribution pattern on WSI. **Conclusions**: FNA is not only feasible for sampling leukocytes from reactive, patient-derived ALNs, but also identifies immune-cell profiles that reflect axillary tumour burden in BC. Thus, this technique could be used to risk-stratify BC patients in the future.

## Simple Summary

Assessing if breast cancer has spread to the armpit (‘axillary’) lymph nodes (ALN) is key in directing treatment. Fine needle aspiration (FNA) is already used routinely in the clinic to detect if cancer cells are present within ALN, but here, we show that the technique can be used to evaluate the axillary immune response. Using flow cytometry, we were able to collect and characterise diverse white blood cell (‘leukocyte’) populations from FNA patient samples, including abundant T-cell subpopulations and rarer natural killer and plasmacytoid dendritic cell populations. Importantly, the immune-cell profiles obtained via FNA appear to correlate with the amount of tumour that is present within the ALN chain (‘axillary tumour burden’). Thus, FNA-based immunophenotyping is a viable, minimally invasive approach for risk stratification in breast cancer.

## 1. Introduction

A third of breast cancer (BC) patients present with axillary lymph node (ALN) metastases [1], and these patients have poorer outcomes [2]. If adjuvant systemic therapy is not given, patients with isolated tumour cells (<0.2 mm or <200 cells) or micrometastasis (0.2–2.0 mm) in ALNs have a lower 5-year survival than patients with negative (tumour-free) ALNs [3]. Similarly, BC patients with ≥3 metastatic ALNs have a worse survival than those with one or two [4,5].

Sentinel lymph nodes (SLNs), which identify the region of the axilla where breast fluid drains, are the first (level 1) ALNs to which BC spreads [6,7]. Accurately assessing SLN status is essential to treatment planning. In the United Kingdom (UK), current guidelines [8] recommend that any ALN that looks suspicious on pre-operative imaging are sampled either with fine needle aspiration (FNA) or core biopsy, both of which are highly specific in detecting metastases [9,10]. While core biopsy is known to be more sensitive than FNA in identifying axillary tumour deposits, it also carries a higher risk of complications. Thus, FNA is preferred for smaller nodes, particularly if close to a vessel [10]. If these needle biopsy samples are negative, an SLN biopsy (SLNB) is performed for histopathological examination during breast surgery [9,11].

This conservative surgical approach is justified by clinical trials that proved that an ALN clearance (ALNC) is not always necessary. The NASBP B-32 trial showed that in patients with negative SLNs, overall and disease-free survival after SLNB alone was equivalent to an ALNC [12]. The Z0011 trial showed the same for patients with only one or two micrometastatic SLNs [5]. Clinically, however, deciding whether patients with one or two macrometastatic (>2 mm) ALNs require a completion clearance remains contentious [13]. Recently, the INSEMA and SOUND trials have challenged if ALN surgery is necessary at all in early-stage BC (T1 and/or T2 with a clinically negative axilla) by showing that omitting ALN surgery is non-inferior to SLNB when assessing disease-free survival [14,15]. Additionally, the emergence of checkpoint inhibitors, together with the understanding that the immune system regulates tumour progression, has prompted further research into how/when it is safe to omit an SLNB in patients who are not at risk of axillary spread.

The LN is an immunological hub that surveys for pathogens and immune threats, and its composition reflects the pathophysiology of the tissues it drains [16]. In pre-metastatic LN, alterations in B- and T-cell localisation and relative abundance of T-cell subsets are thought to precede the arrival of cancer cells [17]. ALN immunophenotype has also been shown to change in advanced BC patients [18], and in murine metastatic nodes, T cells can move towards an effector/memory and exhausted phenotype [19]. Thus, it may be beneficial to look beyond the tumour and its metastases into the LN response itself to identify immunological signatures that predict disease progression.

While FNA is used diagnostically to detect metastatic tumour cells in ALNs, these samples also contain immune cells. Furthermore, unlike core biopsies which remove intact tissue for fixation and histopathological examination, the FNA technique dissociates cells, facilitating a broader range of analyses including flow cytometry. Using FNA with downstream single-cell RNA-sequencing, Provine et al. detected T cells, B cells, natural killer (NK) cells, monocytes, plasmacytoid dendritic cells (pDCs), and conventional dendritic cells in cervical LN [20]. Similarly, in non-small cell lung carcinoma patients, FNA sampling identified site-specific T-cell changes, showing significantly fewer CD4^+^, and more regulatory, T-cells in tumour-draining versus non-tumour draining nodes [21]. Finally, immunoprofiling of joint-draining inguinal LN FNA samples revealed significant differences in B- and CD4^+^/CD8^+^ T-cell composition between healthy volunteers, patients with early rheumatoid arthritis (RA), and individuals at risk of developing RA [22,23,24]. This suggests that FNA immunoprofiling can be used in early disease to predict outcome/risk, but this has yet to be tested in BC.

Here, we sought to determine if FNA with downstream flow cytometry can be used to comprehensively immunoprofile BC-patient-derived ALNs and stratify patients according to axillary tumour burden. We confirm that FNA are rich in, and can detect, diverse immune populations in reactive, patient-derived ALNs. Furthermore, preliminary data suggests that FNA can detect distinct immune changes between patients with one or two metastatic ALNs.

## 2. Materials and Methods

### 2.1. Patients

ALN tissue samples were collected from BC patients (18–95 years old) undergoing a routine ALNC at King’s College Hospital through the Breast Cancer Immunity, Drug, and Gene study (REC reference: 24/NW/0079). Informed consent was obtained from all participants (*n* = 10; Table 1). Samples were de-identified before being released to researchers to ensure blinded data analysis.

### 2.2. FNA of Patient-Derived ALNs

ALNs were dissected out of the ALNC specimen by a Histopathologist (AML/KN) at room temperature within one hour of resection. One to six ALNs (reactive or metastatic) per patient underwent FNA. While applying a vacuum to a 1 mL syringe pre-filled with 500 µL phosphate-buffered saline (PBS), the attached 23G needle was inserted the ALN for single-pass, multiregional sampling before being withdrawn. The PBS was expelled and the syringe then was re-flushed with 500 µL of PBS. Cells were counted manually post-sampling using a haemocytometer. Red blood cells within samples were lysed during fixation for flow cytometry. Samples were kept on ice until staining.

### 2.3. Murine LN Sampling

Charles River female wild-type C57BL/6 mice (*n* = 6) were housed under pathogen-free conditions with a 12 h day/night cycle, with free access to food and water. Experiments were performed under Project Licence PP2472580. After culling, inguinal LNs were removed. FNAs were acquired by inserting a 25G needle, attached to a 1 mL syringe pre-filled with 500 µL, into excised nodes (*n* = 3). The needle was twisted while simultaneously pulling on the syringe plunger during a single pass. Fluid was then expelled from the syringe. Whole inguinal LNs (*n* = 3 per condition) were enzymatically digested with Type 1 collagenase or collagenases A&D (both 1 mg/mL) and DNase (0.1 mg/mL) for 30 min at 37 °C at 500 rpm. Suspensions were pipetted at 15 min to aid digestion. Reactions were stopped with 20 mM ethylenediaminetetraacetic acid. Cells were passed through a 100 µm cell strainer which was subsequently washed with PBS. For mechanical disruption, whole inguinal LNs (*n* = 3) were teased apart using 25G needles on a 100 µm cell strainer. The LNs were mashed with the end of a 1 mL syringe plunger into a single-cell suspension on the strainer; both were then rinsed with 10 mL of PBS. Only two LNs had enough cells to assess cell viability and only one could be immunophenotyped.

### 2.4. Flow Cytometry

Suspensions were centrifuged at 500 g for 5 min and washed with PBS, then stained with Zombie UV™ Live Dead dye (Biolegend, San Diego, CA, USA) at a ratio of 1:500 in PBS for 30 min in the dark at room temperature. Cells were then washed with FACS buffer (2% foetal bovine serum (FBS) in PBS) and resuspended in blocking antibody at a ratio of 1:33 (human) or 1:500 (mouse) for 15 min at room temperature (human) or 4 °C (mouse). Chemokine receptor antibodies were added to samples at a ratio of 1:200 and incubated at 37 °C for 30 min before remaining antibodies (Tables S1 and S2) were added at a ratio of 1:200 and incubated for 1 h at 4 °C. Samples were washed and fixed (FoxP3/Transcription Factor Fixation/Permeabilisation, eBioscience™, San Diego, CA, USA) for 15 min at room temperature which lysed any red blood cells. Cells were then permeabilised and incubated with blocking antibody in permeabilisation buffer (FoxP3/Transcription Factor Fixation/Permeabilisation, eBioscience™) for 15 min at room temperature. Intracellular antibodies were added at a ratio of 1:200 for 30 min at 4 °C. Samples were then washed and resuspended with FACS buffer and read on either CytoFLEX (Beckman Coulter, Brea, CA, USA) or LSRFortessa (BD Biosciences, Milpitas, CA, USA). During analysis, FNAs that acquired fewer than 1 × 10^4^ CD45^+^ cells were excluded from immunophenotyping analysis. Data was analysed using FlowJo 10.10.0. A native platform within FlowJo was used to generate t-SNEs.

### 2.5. IHC

Formalin-fixed and paraffin-embedded reactive ALN tissue sections (3 µm thick; *n* = 8 patients) were immunostained using automated staining protocols with either CD4 (1:80), CD56 (1:50), or CD123 (1:100) antibodies after heat-induced epitope retrieval in pH9 buffer. Automated staining was completed using the Optiview Kit on Roche Benchmark Ultra for CD4 and CD56 or BOND Compact Polymer Detection kit on Leica Bond III for CD123.

### 2.6. Image Analysis

Whole-slide images were captured using the Glissando Desktop Scanner (Objective Imaging). The proportion of CD4^+^, CD56^+^, and CD123^+^ cells was determined using QuPath software V.0.6.0 by quantification of the whole ALN (Figure S7) [25]. The pixel classifier using a hematoxylin stain threshold was used to exclude background areas. A second pixel classification for DAB staining was used on CD123 images to exclude weakly positive HEVs and sinuses, confirmed with a manual adjustment by a Histopathologist (AML/KN). IHC-positive cells were calculated as a percentage of all cell nuclei in the selected ALN areas using the positive-cell detection tool. FNA-positive cells were calculated as a percentage of all single cells and compared to IHC-positive cells as a percentage of all cell nuclei (CD4^+^ and CD56^+^) or cell nuclei outside of HEVs and sinuses (CD123^+^) in tissue sections.

### 2.7. Statistical Analysis

All statistical analysis was performed on GraphPad Prism V 10.4.1. For parametric data, two groups were compared by one-way ANOVA; >two groups were analysed by two-way ANOVA. Pairs of unmatched, non-parametric data were compared using the Mann–Whitney test. The Kruskal–Wallis test was used for multiple comparisons of non-parametric data. A *p*-value of ≤0.05 was considered significant.

## 3. Results

### 3.1. FNA Samples the Same Immune Cells as Whole-LN Digestion

**Figure 1:**
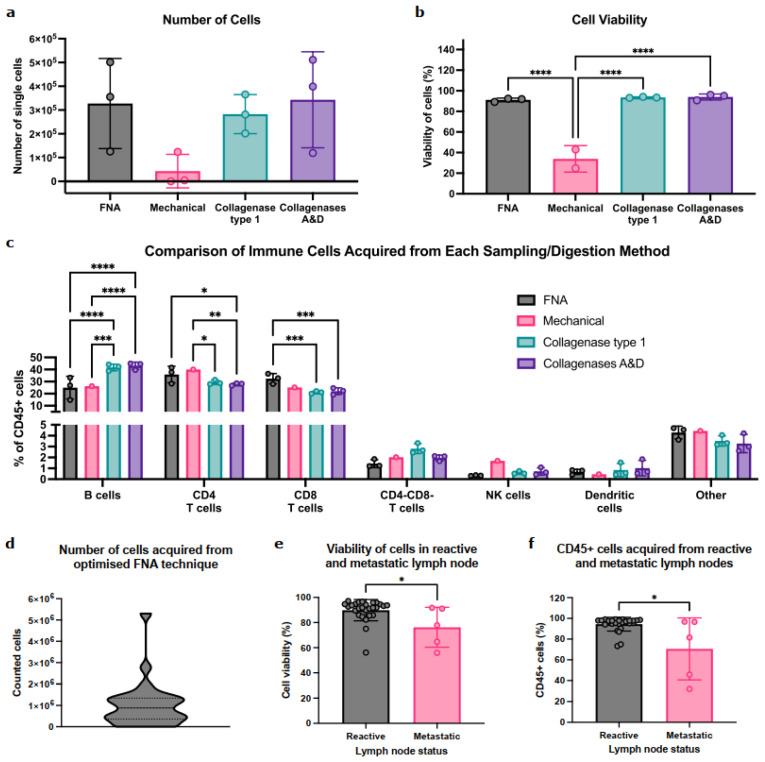
FNA reproducibly captures diverse immune-cell populations in mice and is feasible in patient-derived samples. (**a**) Comparison of the number of cells acquired between LN sampling methods in C57/BL6 female mice, quantified by flow cytometry. Data presented as mean ± SD, each point represents one inguinal LN; *n* = 3 per group. One-way ANOVA was used to determine statistical significance. (**b**) Quantification of cell viability across LN sampling methods in mice. Data presented as mean ± SD, each point represents percentage viability of cells from one inguinal LN; *n* = 3 per group apart from mechanical digestion where *n* = 2. One-way ANOVA was used to determine statistical significance. (**c**) Quantification of immune-cell abundance obtained between each sampling method in mice. Data presented as mean ± SD, each point is one inguinal LN; *n* = 3 per group apart from mechanical digestion where *n* = 1. Two-way ANOVA used to determine statistical significance. (**d**) Distribution of the number of cells acquired from the optimised FNA technique in patient samples. Cells were counted manually on a haemocytometer immediately post sampling; *n* = 27 ALN. (**e**) Comparison of cell viability between reactive and metastatic patient-derived ALNs measured by flow cytometry. Data presented as mean ± SD. Reactive ALN: *n* = 27, metastatic ALN: *n* = 5. Mann–Whitney test was used to determine significance. (**f**) Comparison of the percentage of CD45^+^ cells acquired between reactive and metastatic patient-derived ALNs determined by flow cytometry. Data presented as mean ± SD. Reactive ALN: *n* = 27, metastatic ALN: *n* = 5. Mann–Whitney test was used to determine significances. (* *p* < 0.05, ** *p* < 0.01, *** *p* < 0.001, and **** *p* < 0.0001).

To first determine if FNA captured the immune diversity seen in standard whole-node processing methods, we compared the contents of FNA samples to whole-LN-cell suspensions (i.e., mechanical disruption or enzymatic digestion with either Type 1 collagenase or collagenases A&D) using murine LNs. The number and viability of immune cells in FNA samples were comparable to those obtained with collagenase digestion (Figure 1a,b and Figure S1); mechanical digestion was the least efficient (*n* = 2 (zero cells collected from one LN)).

Importantly, FNA sampled all the immune-cell (CD45^+^) populations within the node (Figure 1c). B cells were abundant in FNA, albeit at lower levels than collagenase digestion methods. Conversely, FNA enriched for CD4^+^/CD8^+^ T-cell populations. Importantly, rarer populations (CD4^−^CD8^−^ T cells, NK cells, and/or DC) were detected in similar proportions by both digestion and FNA. Interestingly, while enzymatic digestion identified positive and negative cell populations, these were dimmer and less distinct than in FNA, indicating that FNA sampling negated the effect of enzymatic digestion on cell surface epitopes (Figure S2).

Thus, FNA can acquire abundant, diverse immune populations from murine LNs without impacting cell viability or epitope availability.

### 3.2. FNA Is Feasible and Identifies Diverse Immune Populations in Patient-Derived ALN

We then tested feasibility in patient ALNs. On average, FNA acquired 1 × 10^6^ cells per reactive ALN (Figure 1d). Interestingly, the presence of ALN metastasis significantly decreased cell viability (Figure 1e; *p* = 0.02) and significantly fewer immune cells (CD45^+^) were acquired from metastatic ALNs (Figure 1f; *p* = 0.03).

**Figure 2:**
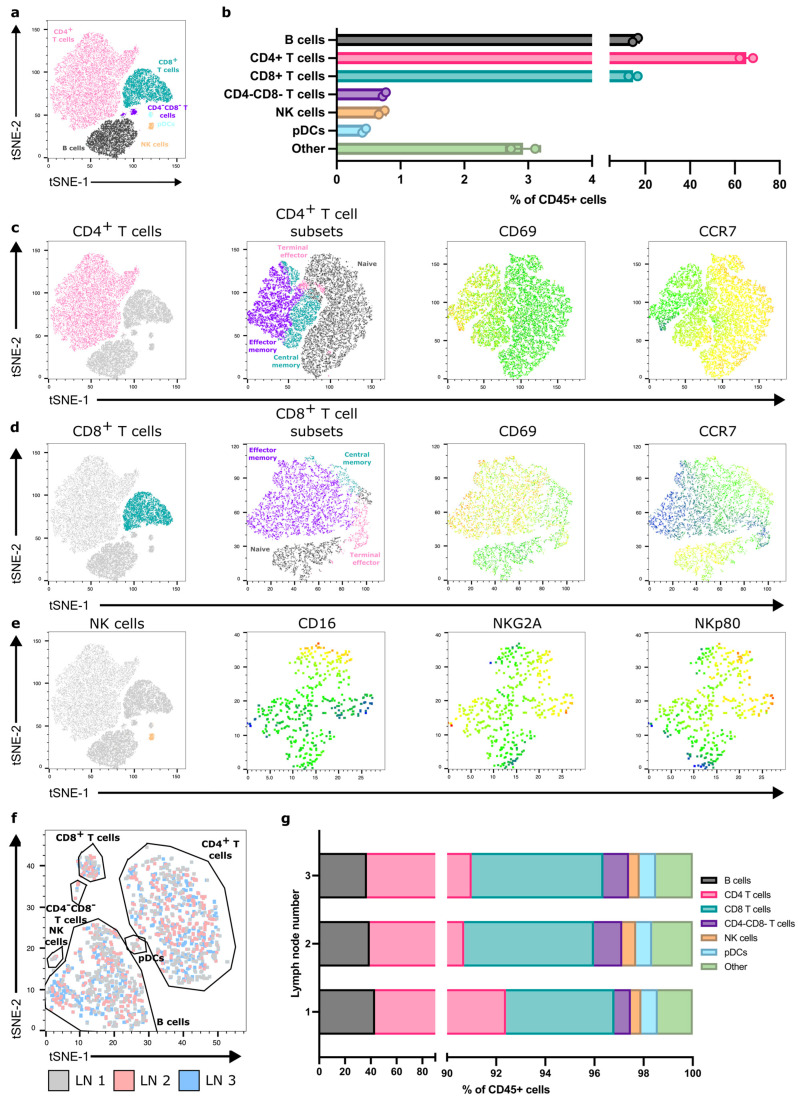
FNA consistently acquires diverse immune populations across reactive, patient-derived ALNs. (**a**) Representative flow cytometry derived t-SNE on CD45^+^ cells from one patient sample showing the major immune-cell populations acquired. (**b**) Quantification of immune-cell populations detected within FNA samples from the same patient. Data presented as mean ± SD. Each point represents one ALN; *n* = 2. (**c**) Representative t-SNE showing CD4^+^ T-cell subsets detected within samples (naïve, black; terminal effector, pink; effector memory, purple; central memory, aqua) and CD69 and CCR7 expression within these subsets (colour scale—blue, low expression; red, high expression); (**d**) Representative t-SNE showing CD8^+^ T-cell subsets and CD69 and CCR7 expression; and (**e**) NK cell surface receptor expression identified through FNA sampling. (**f**) Representative t-SNE generated from flow cytometry data depicting overlapping immune-cell clustering from each of the three ALNs collected from the same patient. (**g**) Flow cytometry quantification of proportions of immune-cell populations between each of the three ALNs from the same patient.

With FNA, we could identify and quantify the major immune-cell populations within reactive ALNs (Figure 2a,b and Figure S3A; gating strategy shown in Figure S4; *n* = 9 patients). As expected, most were T and B cells. We could also detect and characterise rarer populations, e.g., pDCs and NK cells. Moreover, FNA discriminated between immune-cell subpopulations within each cluster (Figure 2c–e), detecting naïve, terminal effector, central memory, and effector memory T-cell subsets, alongside identifiers of functional and activation states. Finally, we compared immune-cell proportions between patients and demonstrated limited inter-nodal variability (Figure S3B).

### 3.3. One Reactive ALN May Reflect the Immune Status of the Axilla

Typically, one to four ALNs are collected during an SLNB, and more during an ALNC [23]. Therefore, we assessed the degree of variability in immune-cell composition between individual, reactive ALNs from each patient. T-SNE analysis showed that all reactive ALNs from the same patient clustered consistently, irrespective of where the node is in the chain (Figure 2f,g and Figure S5). Thus, it appears, in this feasibility cohort, that all reactive ALNs within a patient have a similar immune profile.

### 3.4. FNA and Immunohistochemistry of the Same ALN Show Similar Immune Profiles

Immunohistochemistry (IHC) is used routinely in diagnostic practice to immunophenotype patient-derived ALNs. Therefore, we compared FNA sampling with IHC of an abundant cell population (CD4^+^ T cells), as well as rarer cell populations (CD56^+^ NK cells or CD123^+^ pDCs), from the same ALN to ensure that FNA sampling was representative (*n* = 8 patients).

**Figure 3:**
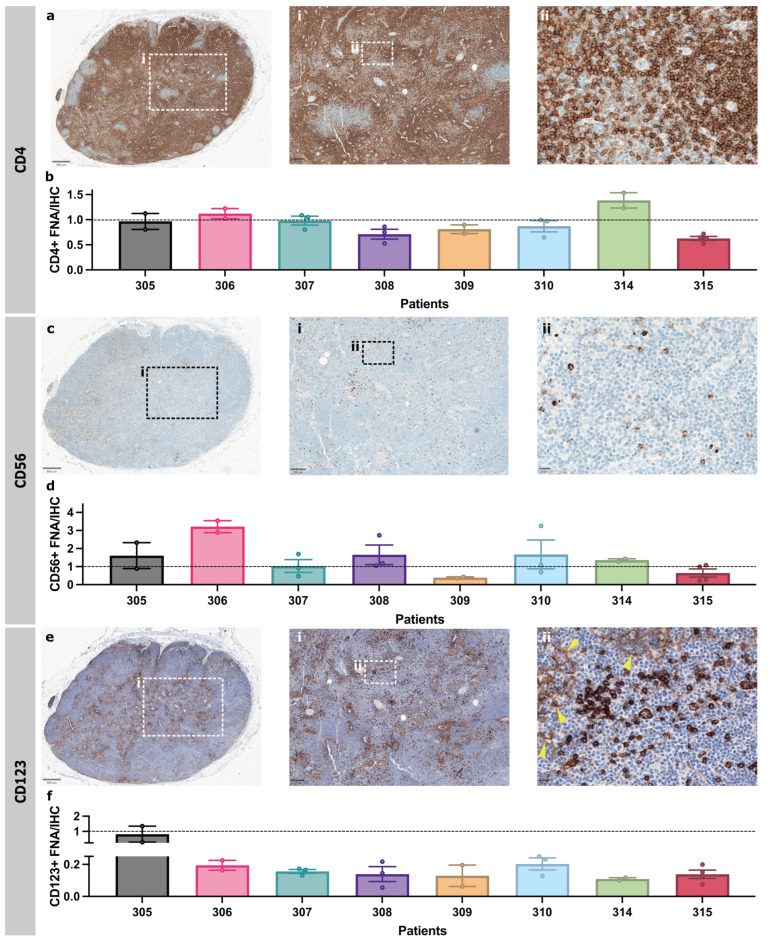
FNA and IHC of the same ALN identify similar abundant and scarce immune-cell types within patient-derived ALN. (**a**) Representative photomicrographs of patient-derived ALNs immunostained with CD4 (birds-eye view, ×50 (inset i) and ×400 (inset ii) magnification. (**b**) Proportion of CD4^+^ cells in FNA sample compared to that in IHC images. (**c**) Representative photomicrographs of patient-derived ALNs immunostained with CD56 (birds-eye view, ×50 (inset i) and ×400 (inset ii) magnification. (**d**) Proportion of CD56^+^ cells in FNA sample compared to that in IHC images. (**e**) Representative photomicrographs of patient-derived ALNs immunostained with CD123 (birds-eye view, ×50 (inset i) and ×400 magnification (inset ii). Yellow arrowheads denote weaker HEV staining. Samples were counterstained with Haematoxylin for nuclear identification. (**f**) Proportion of pDCs (CD4^+^HLA-DR^+^) in FNA sample compared to proportion of that in IHC images. All bars are mean ± SD. Each point represents one ALN.

IHC quantification and FNA identified similar proportions of CD4^+^ T cells (Figure 3a,b). NK cells showed strong, granular cytoplasmic CD56 staining and were singly dispersed throughout the paracortex (Figure 3c). There was some variation in the FNA proportions of NK cells compared to IHC-based image analysis from individual patients—FNA generally enriched NK cells, but only patient 306 was a significant outlier (Figure 3d). pDCs showed strong cytoplasmic and membranous CD123 staining and were patchily distributed as clusters throughout the paracortex (Figure 3e). High endothelial venules (HEVs) and sinus macrophages were weakly CD123 positive. These were excluded during quantification by thresholding for staining intensity. FNA samples contained fewer pDCs than in IHC (Figure 3f). Overall, FNA still captured these rarer cell populations, and detected variations in cell surface protein expression in a representative and interpretable fashion.

### 3.5. Immune-Cell Proportions Correlate with Axillary Tumour Burden

**Figure 4:**
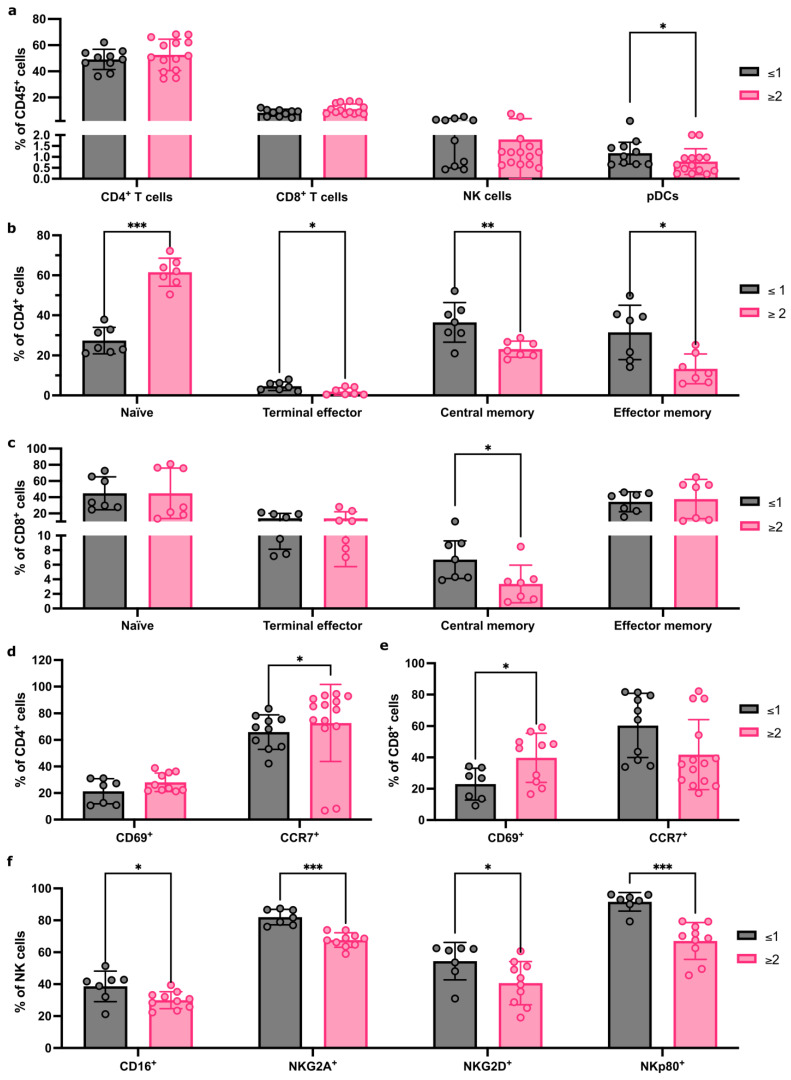
FNA captures immune-cell population changes as tumour burden increases in the axilla. (**a**) Flow cytometry quantification of major immune population abundance within FNA samples from patients with ≤1 or ≥2 metastatic ALNs. (**b**) Flow cytometry quantification of changes to CD4^+^ T-cell subpopulation abundance between patients with ≤1 or ≥2 metastatic ALNs; Naïve, terminal effector, central memory, and effector memory subsets presented as % of total CD4 T cells. (**c**) Quantification of CD8^+^ T-cell subsets as for (**b**) between patients with ≤1 or ≥2 metastatic ALNs. (**d**,**e**) Flow cytometry quantification of markers of activation status, CD69 and CCR7, in CD4^+^ (**d**) and CD8^+^ (**e**) T cells comparing patients with ≤1 or ≥2 metastatic ALNs. Data represented as % of parent population (CD4 or CD8, respectively). (**f**) Quantification of the effect of total number of metastatic ALNs on the frequency of NK cells expressing CD16, NKG2D, NKG2A, and NKp80 markers of activation status. Data presented as mean ± SD. ≥2 ALNs: *n* ≥ 7, ≤1 ALNs: *n* ≥ 7, and each point represents an individual ALN. The Mann–Whitney test was used to determine statistical significance. (* *p* < 0.05, ** *p* < 0.01 and *** *p* < 0.001).

Finally, we tested if FNA sampling could identify immune differences in reactive ALNs from patients with different tumour volumes within their axilla. Patients were stratified into two groups (≤1 or ≥2 metastatic ALNs). There were no proportional differences in CD4^+^ and CD8^+^ T cells or NK cells, but pDCs were significantly decreased in the cohort with ≥2 metastatic ALNs (Figure 4a; *p* = 0.03; gating strategy shown in Figure S6).

CD4^+^ and CD8^+^ T-cell subpopulations differed between the two groups (Figure 4b,c). Naïve CD4^+^ T cells (*p* < 0.001) increased significantly, and CD4^+^ terminal effector (*p* = 0.04), CD4^+^ effector memory (*p* = 0.01), and CD4^+^ central memory T cells (*p* = 0.007) significantly decreased in the group with ≥2 metastatic ALNs. Within CD8^+^ T-cell subsets, central memory T cells decreased significantly in the cohort with ≥2 metastatic ALNs (*p* = 0.01).

We then compared activation receptor CD69 and chemokine receptor CCR7 expression in CD4^+^ and CD8^+^ T cells (Figure 4d,e). A significant increase in CD69-expressing CD8^+^ T cells (*p* = 0.04) and CCR7-expressing CD4^+^ T cells (*p* = 0.04) was detected in patients with ≥2 involved ALNs, which correlates with the increase in naïve CD4^+^ T cells observed.

Finally, we evaluated if there were any changes in NK cell phenotype and activation state between groups (Figure 4f). A significant decrease in CD16 (*p* = 0.05), NKG2A (*p* < 0.001), NKG2D (*p* = 0.03), and NKp80 (*p* < 0.001) expression was seen in patients with ≥2 metastatic ALNs. Together, these data indicate that FNA is sensitive enough to detect distinct immune changes between patients, based on the extent of ALN metastatic disease.

## 4. Discussion

Although SLNB alone is now the standard of care for clinically node-negative BC patients, we have yet to define exactly when the axilla becomes immunosuppressed in early BC, facilitating metastasis. To this end, we have shown that the minimally invasive FNA technique can be used to comprehensively immunophenotype reactive LNs within the axillary chain. Furthermore, since our preliminary data suggests that an immunophenotype ‘snapshot’ reflects the amount of metastatic tumour within the patient’s axilla, this method could potentially be used to risk-stratify patients in the future.

The murine experiments confirmed that FNA sampling was representative of whole-LN composition. Furthermore, enzymatic digestion commonly alters cell surface receptors and mechanical digestion often fails to isolate DC [26,27]. FNA avoided both these pitfalls.

Consistent with previous clinical studies using ultrasound-guided FNA, we acquired on average 1 × 10^6^ cells per FNA from patients [28,29]. Furthermore, our yields were sufficient to comprehensively immunophenotype reactive ALNs. These FNA samples contained a wide range of immune cells, including less abundant populations. As in haematological malignancies [30], FNA acquired more T cells than B cells. However, both B- and T-cell subpopulations were detectable, and functional markers reflected activation state. This carried forward to rarer pDC and NK populations. Even though IHC showed more pDCs than matched FNA samples, the latter could still be quantified. Importantly, our data suggests that one reactive ALN is representative of the entire axilla, i.e., sampling one reactive ALN delineates if the patient is immunocompetent or immunosuppressed. Translationally, this is relevant as the radiologist could pre-operatively FNA on the most accessible reactive ALN under ultrasound guidance in order to risk-stratify patients. We are amending our study ethics to test this in a larger cohort of early BC patients in the near future. Since these patients would still require an SLNB in line with current guidelines, we will be able to directly compare pre-operative FNA to ex vivo FNA and whole-node histology [31]. This will ensure that no prognostic information is lost by shifting focus from quantifying axillary tumour burden per se to functionally measuring the immune response. Ultimately, we hope that repurposing the FNA technique will identify a subset of immunocompetent BC patients in whom axillary surgery could be omitted entirely.

Using IHC, López et al. showed that CD8^+^ T cells and CD68^+^ macrophages significantly increased, but CD123^+^ pDCs significantly decreased, in the reactive ALNs of node-positive, treatment-naïve BC patients [32]. Interestingly, we have also shown that pDCs significantly decrease in the reactive ALNs of BC patients once ≥2 ALNs elsewhere in the axillary chain contain metastatic tumours. This suggested correlation in the proportion of pDCs to axillary tumour burden warrants further investigation in a larger cohort of patients—we plan to do this in the future.

We have also shown that NK cell surface expression of CD16, NKG2A, NKG2D, and NKp80 correlates with the degree of ALN involvement. Using LN fragments, Frazao et al. showed that reactive and metastatic ALNs expressed similar NK receptors, but both NKG2A and CD16 expression were significantly increased on NK cells from tumour-draining ALNs compared to mesenteric nodes from healthy donors, irrespective of whether they contained tumours, in stage IIIA compared to stage II patients [33]. One could argue that LNs from different body sites (in this case, axilla versus abdomen) will always have different immune profiles since they are exposed to different antigens, and therefore mesenteric LNs may not have been a suitable control [16,34]. Moreover, while that study showed that CD16 and NKG2A expression increased in N2–3 versus N1 patients, it did not stratify the N1 cohort based on the number of metastatic ALNs as we have.

T-cell researchers have also only compared reactive ALNs to metastatic ALNs. Vahidi et al. showed an increase in central memory CD8^+^ T cells, but a decrease in naïve CD4^+^ T cells, in metastatic compared to reactive ALN fragments [35]. This differs from our FNA-based data which showed a decrease in central memory T cells (including central memory CD4^+^ T cells), but an increase in naïve CD4^+^ T cells, in patients with ≥2 involved nodes. This again suggests that the presence of tumours in other nodes within the axillary basin influences the immune response in reactive ALNs and is more nuanced than previously thought.

Finally, our study design was restricted by the fact that pre-operative FNA in the UK is currently permitted only to assess suspicious, and not reactive, ALNs. Therefore, we opted to first assess if the technique was feasible ex vivo. We were able to show in these 10 patients that we can comprehensively immunoprofile reactive ALNs using FNA with downstream flow cytometry. Encouragingly, we were also able to quantify statistically significant immunological differences between two cohorts of BC patients based on axillary tumour burden. We now aim to validate these findings in a larger cohort of BC patients, as outlined above.

## 5. Conclusions

In summary, we have shown that FNA, which is minimally invasive, can reproducibly identify immune-cell changes in reactive, patient-derived ALNs in BC. Furthermore, since FNA is already used to diagnose BC nodal metastasis pre-operatively, it could easily be repurposed to sample the most accessible, reactive ALNs to delineate the patient’s locoregional immune response. Finally, we have shown through a preliminary cohort of BC patients that distinct immunological changes may reflect axillary tumour burden. If this bears out in future studies, we believe that this technique could be harnessed to risk-stratify patients with early BC in the future.

## Figures and Tables

**Table 1:** Clinical characteristics for all patients.

Characteristic	Number of Patients (%)
**Age**	
<50	3 (30%)
≥50	7 (70%)
**Treatment**	
Naïve (Rx)	6 (60%)
NACT	4 (40%)
**Molecular subtype**	
ER^+^	10 (100%)
HER2^+^	4 (40%)
**Number of reactive LN**	
0	1 (10%)
≥1	9 (90%)
**Number of involved LN**	
≤1	3 (30%)
≥2	7 (70%)

## Data Availability

Data in this study can be made available from the corresponding authors on request.

## Supplementary Materials

The following supporting information can be downloaded at https://www.mdpi.com/article/10.3390/cancers18020251/s1. Figure S1: Gating strategy to identify murine immune-cell populations in inguinal LN; Figure S2: FNA preserves the integrity of cell surface markers CD3, CD19, CD4, and CD8; Figure S3: FNA acquires major immune-cell populations across all patient-derived reactive ALNs; Figure S4: Gating strategy to define major immune-cell populations in FNA samples from breast-cancer-patient-derived reactive ALNs; Figure S5: One reactive LN is consistently representative of all reactive LNs in a patient’s axilla; Figure S6: Gating strategies to define immune-cell subsets and receptor expression; Figure S7: Quantification of CD4^+^, CD56^+^, or CD123^+^ cells in IHC images; Table S1: Anti-mouse antibodies used to compare FNA sampling to whole-lymph-node digestion; Table S2: Anti-human antibodies used to define immune-cell populations from patient ALNs.

## Abbreviations

The following abbreviations are used in this manuscript:

ALN	Axillary lymph nodes
ALNC	Axillary lymph node clearance
BC	Breast cancer
DC	Dendritic cell
FNA	Fine needle aspiration
HEV	High endothelial venule
IHC	Immunohistochemistry
LN	Lymph node
NK	Natural killer
PBS	Phosphate-buffered saline
pDC	Plasmacytoid dendritic cell
RA	Rheumatoid arthritis
SLN	Sentinel lymph nodes
SLNB	Sentinel lymph node biopsy
WSI	Whole-section immunohistochemistry
