# Supplementary material for: Immune Profiling the Axilla with Fine Needle Aspiration Is Feasible to Risk-Stratify Breast Cancer
Source: Cancers (Basel). 2026 Jan 14;18(2):251. doi: 10.3390/cancers18020251 (PMC12838821; doi:10.3390/cancers18020251)
Supplement: Supplementary file 1 [file cancers-18-00251-s001.zip › cancers-4078407-supplementary.pdf]

Supplementary Materials

Supplementary Figures

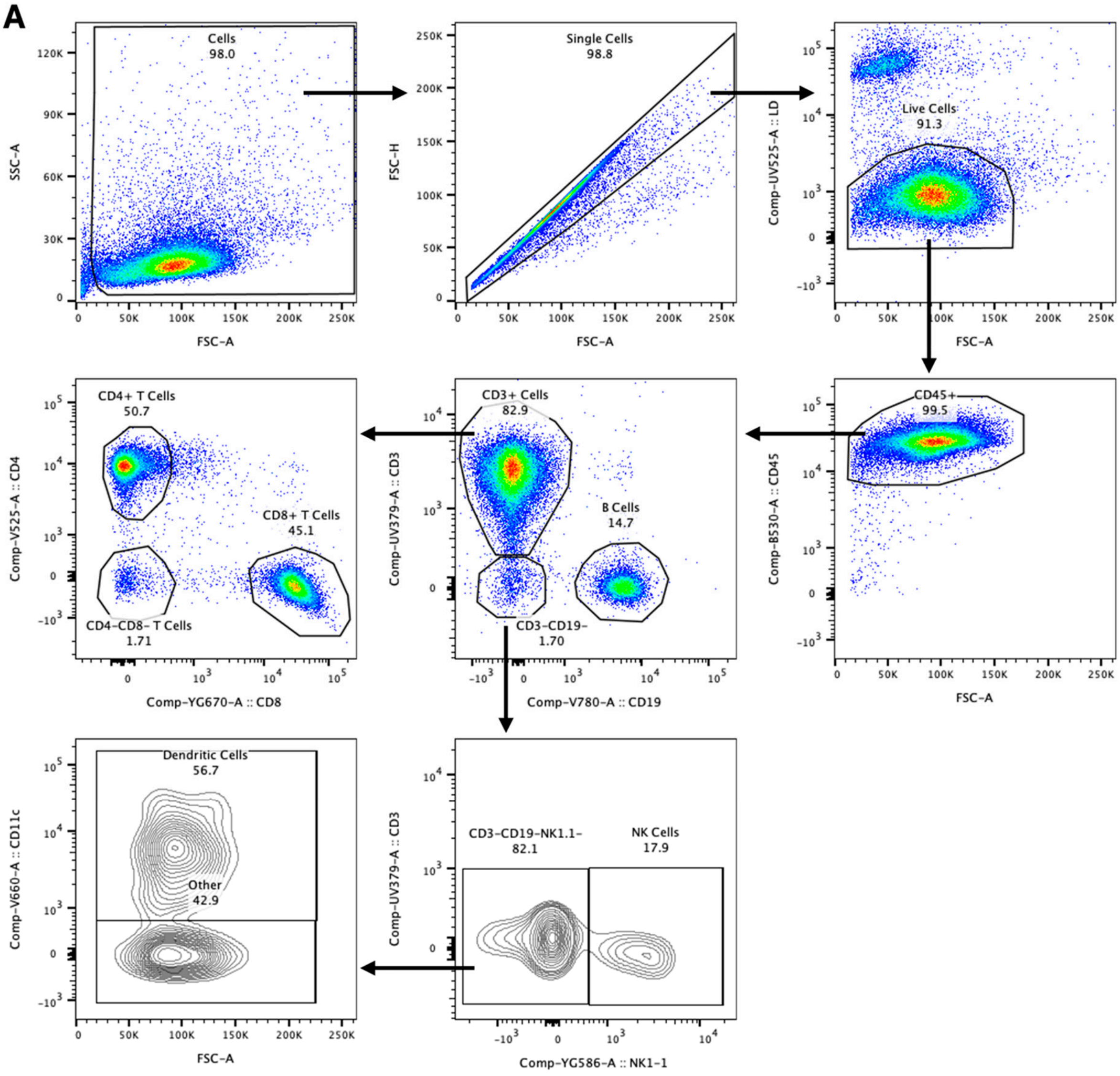

Figure S1. Gating strategy to identify murine immune cell populations in inguinal LN.

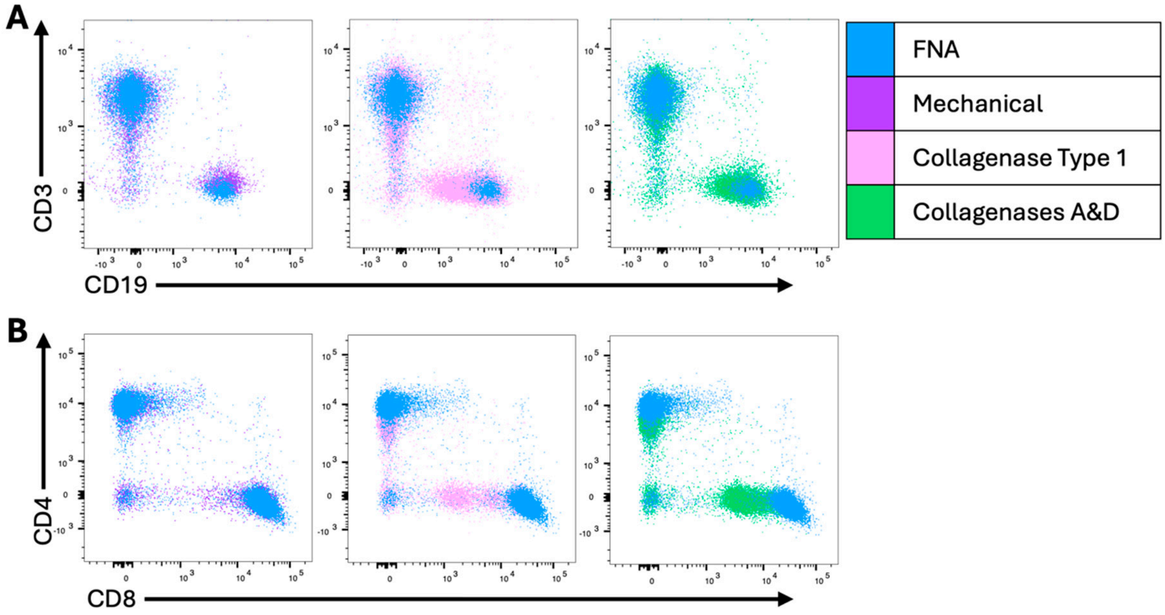

Figure S2. FNA preserves the integrity of cell surface markers CD3, CD19, CD4 and CD8. Murine inguinal LN were sampled/digested using either FNA, mechanical digestion, collagenase type 1 or collagenases A&D. FNA (blue) is overlaid on mechanical (purple), collagenase type 1 (pink) or collagenases A&D (green). (**A**) Dot plots showing staining of CD3 and CD19 on $CD45^+$ immune cells. (**B**) Dot plots showing staining of CD4 and CD8 on $CD45^+CD3^+$ immune cells.

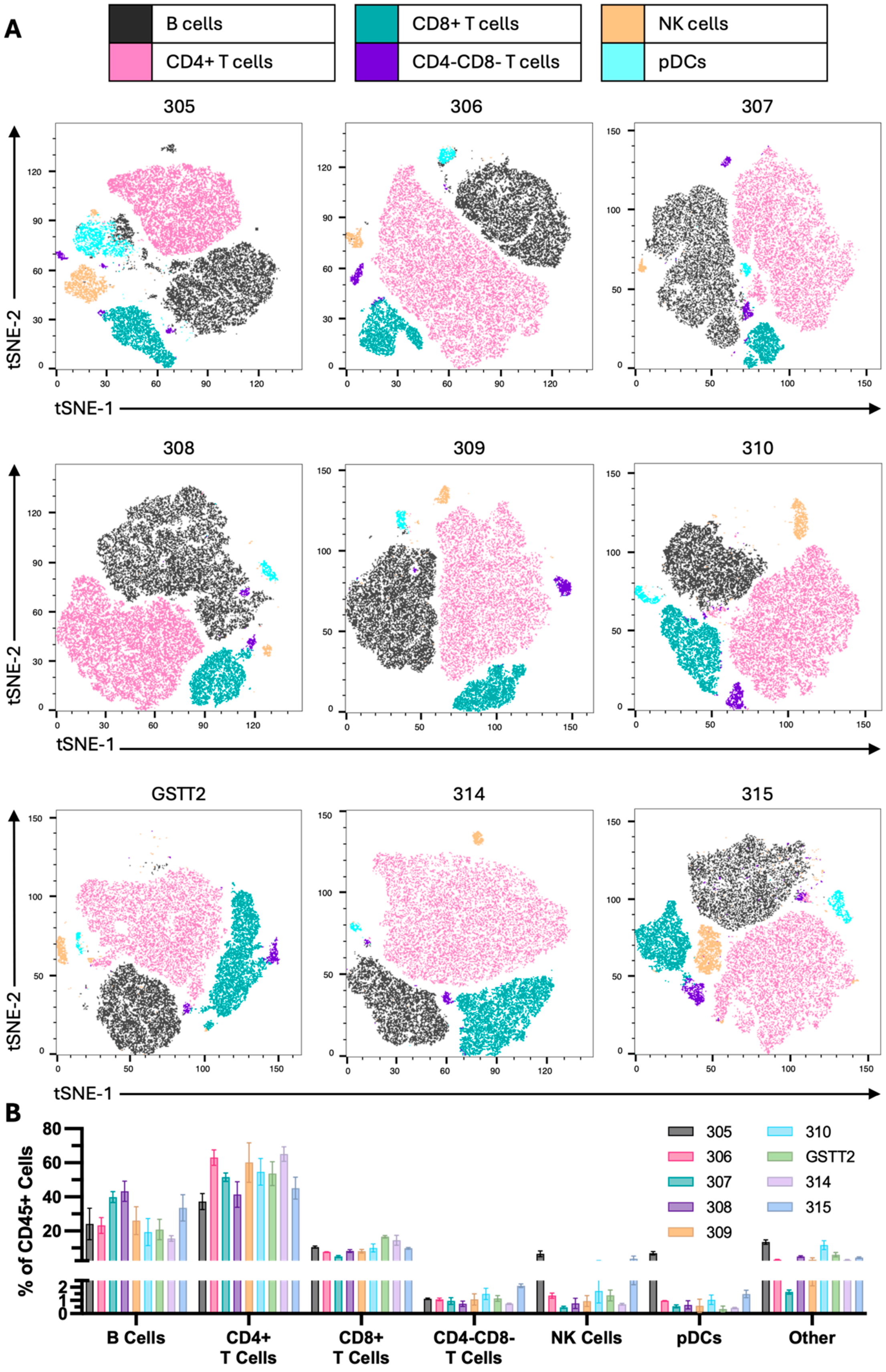

Figure S3. FNA acquires major immune cell populations across all patient-derived reactive ALN. (**A**) Each panel is a representative tSNE from one reactive ALN from each of nine patients, showing the immune cell populations collected in the FNA. (**B**) Graph showing inter-patient variability of the immune cell proportions collected from ALN using FNA.

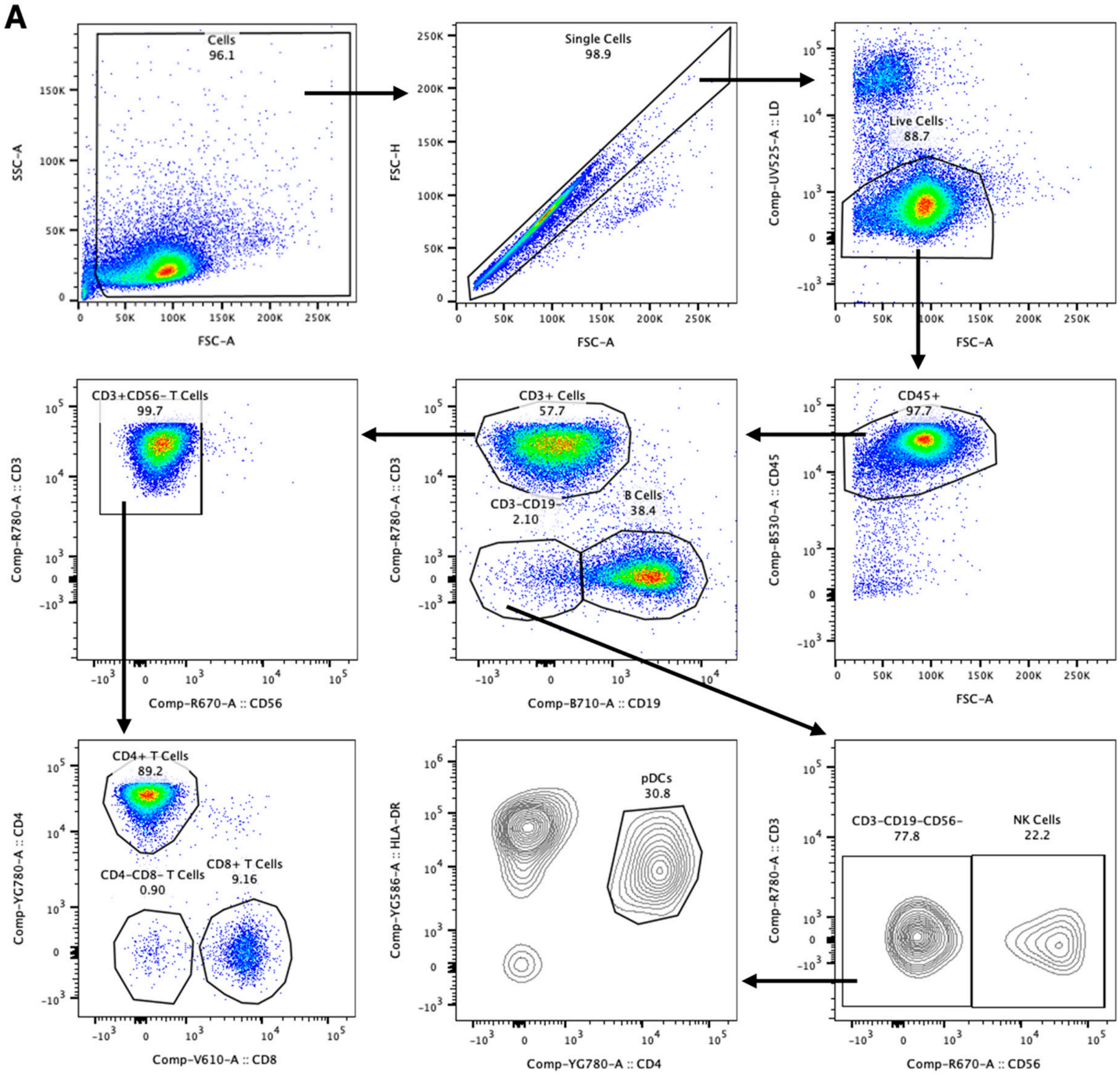

Figure S4. Gating strategy to define major immune cell populations in FNA samples from breast cancer patient-derived reactive ALN.

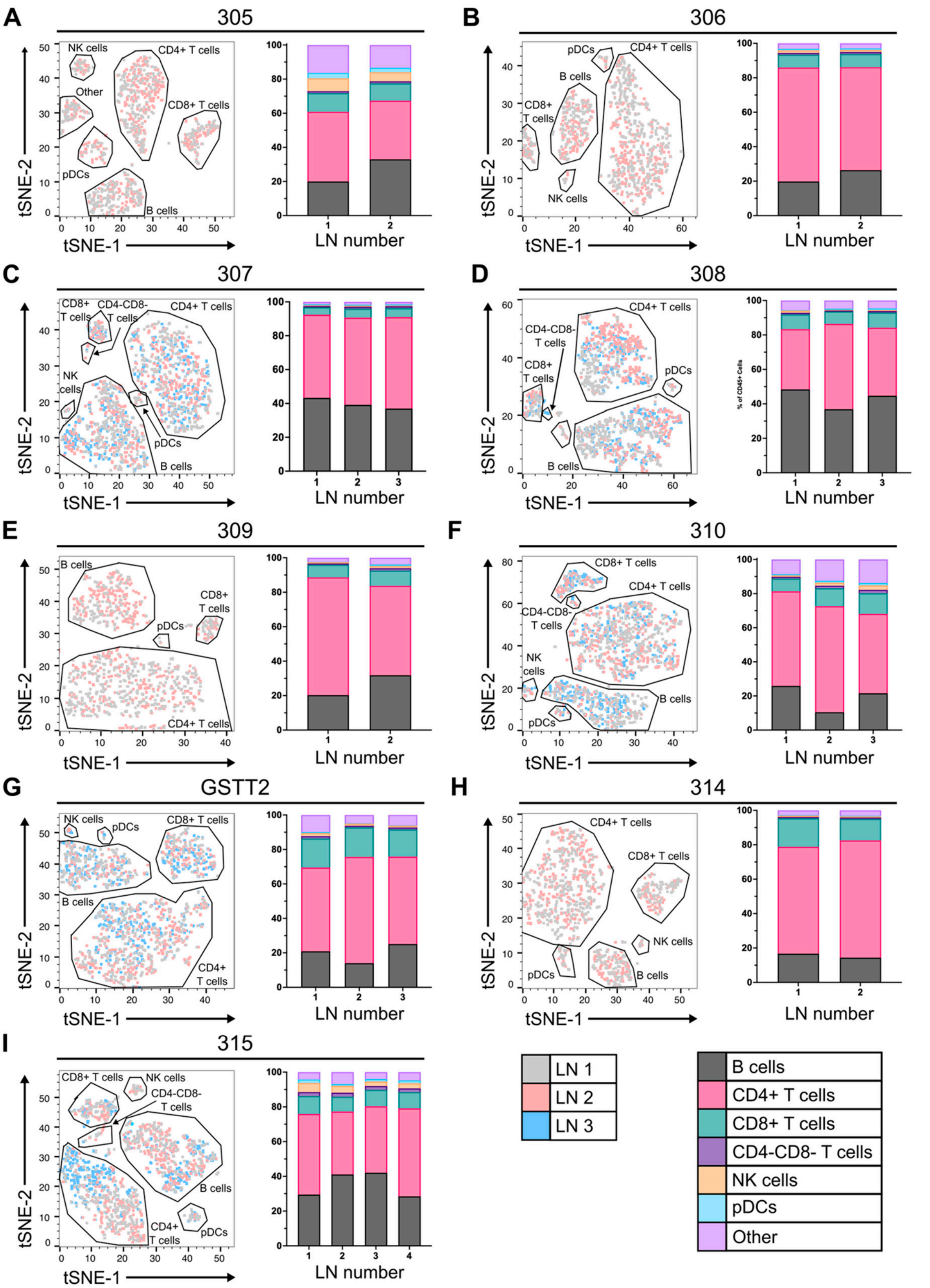

Figure S5. One reactive LN is consistently representative of all reactive LN in a patient's axilla. (**A – I**) represent each of nine patients that had at least two reactive LNs sampled using FNA in the axilla. TSNEs represent clustering of immune cells from reactive ALN sampled from the same patient. Stacked bar charts show the proportions of each immune cell population from each ALN.

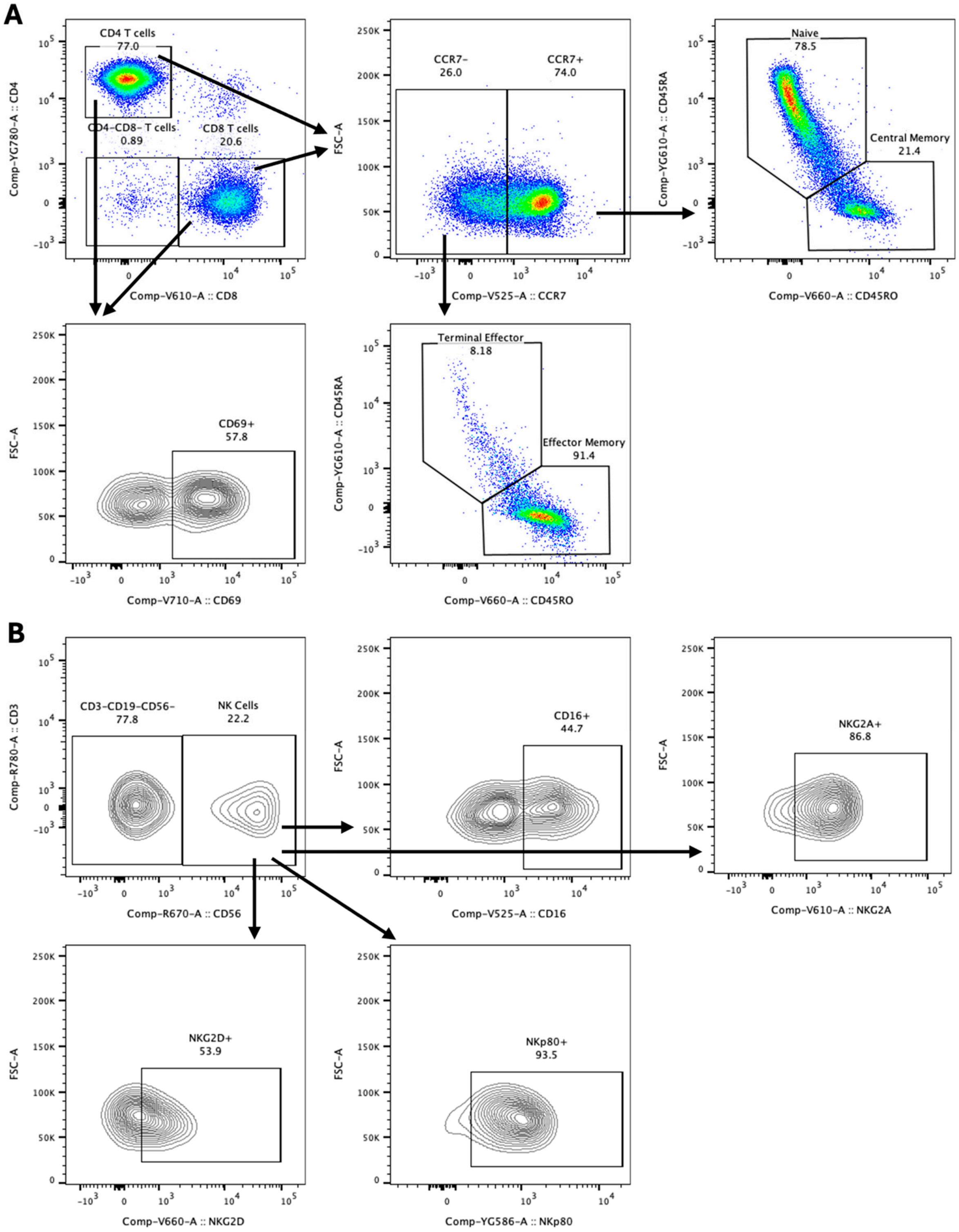

Figure S6. Gating strategies to define immune cell subsets and receptor expression. (**A**) Gating strategy to define CD4+ and CD8+ T cell subsets (naïve, terminal effector, central memory and effector memory), and CD69 and CCR7 expression. (**B**) Gating strategy to determine receptor expression (CD16, NKG2A, NKG2D and NKp80) on NK cells.

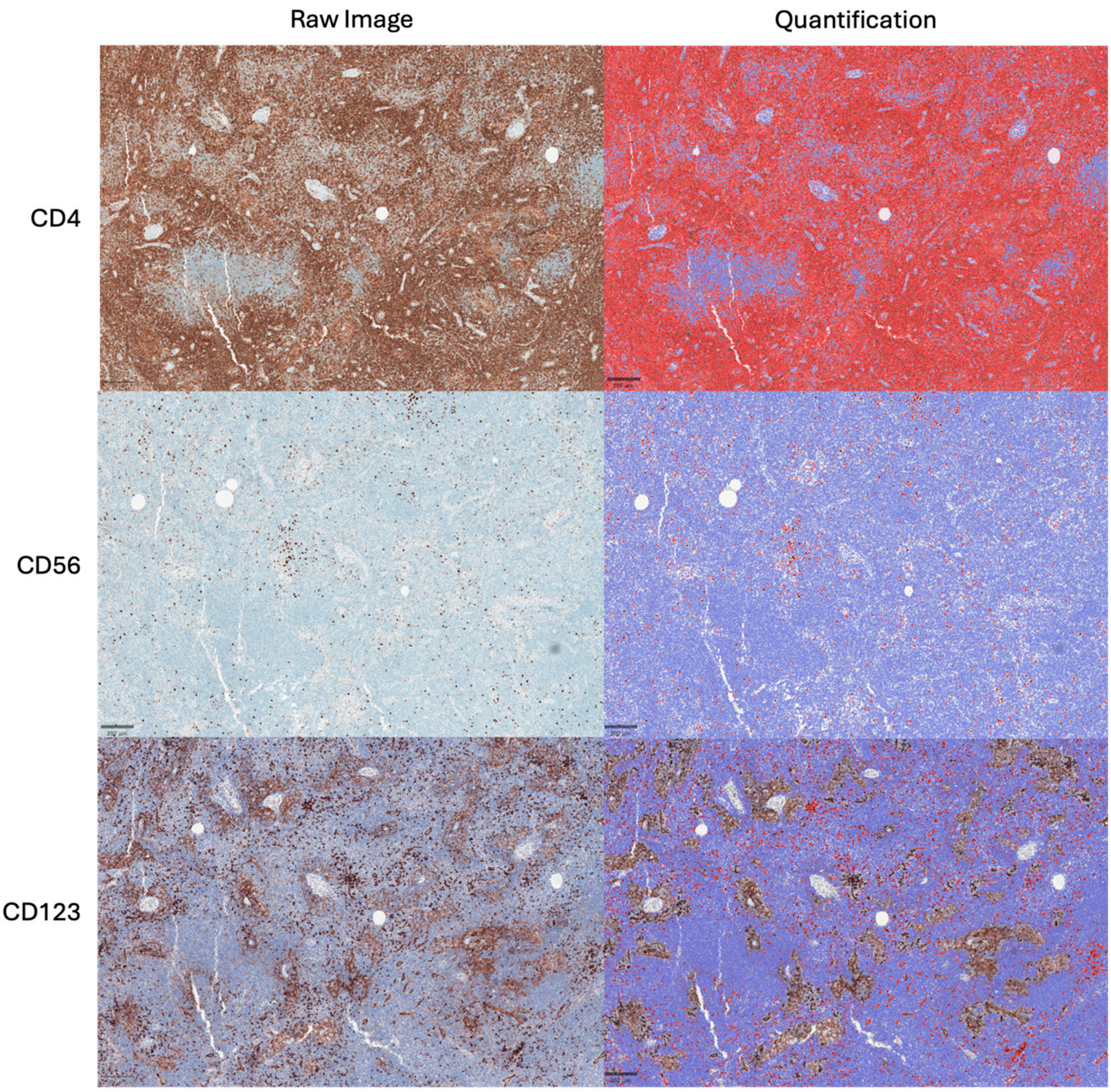

Figure S7. Quantification of CD4⁺, CD56⁺ or CD123⁺ cells in IHC images. Red (positive) and blue (negative). Scale bars 200 µm.

Table S1. Anti-mouse antibodies used to compare FNA sampling to whole-lymph node digestion.

Target	Clone	Fluorochrome	Supplier
Zombie UV Fixable Viability Dye	N/A	Zombie UV	Biolegend
CD3e	145-2C11	BUV395	BD Horizon™
CD4	RM4-5	BV510	Biolegend
CD8a	53-6.7	PE/Cyanine5	Biolegend
CD11b	M1/70	APC/Cyanine7	Biolegend
CD11c	N418	BV650	Biolegend
CD16/32	93	N/A	Biolegend
CD19	6D5	BV785	Biolegend
CD45	30-F11	FITC	Biolegend
F4/80	BM8	PE/Cyanine7	Biolegend
NK-1.1	PK136	PE	Biolegend

Table S2. Anti-human antibodies used to define immune cell populations from patient ALN.

Target	Clone	Fluorochrome	Supplier
Zombie UV Fixable Viability Dye	N/A	Zombie UV	Biolegend
Human TruStain FcX™ Block	N/A	N/A	Biolegend
CD3	HIT3a	APC/Cyanine7	Biolegend
CD4	SP35	None	Roche
CD4	SK3	PE/Cyanine7	Biolegend
CD8a	RPA-T8	BV605	Biolegend
CD16	3G8	BV510	Biolegend
CD19	SJ25C1	PerCP/Cyanine5.5	Biolegend
CD19	SJ25C1	APC/Cyanine7	Biolegend
CD45	HI30	FITC	Biolegend
CD45RA	HI100	PE/Dazzle	Biolegend
CD45RO	UCHL1	BV650	Biolegend
CD56	123C3	None	Dako
CD56	5.1H11	APC	Biolegend
CD69	FN50	BV711	Biolegend
CD123	BR4MS	None	Cell Signaling Technology
CD197 (CCR7)	G043H7	PE/Dazzle	Biolegend
CD197 (CCR7)	G043H7	BV510	Biolegend
HLA-DR	L243	PE	Biolegend
NKG2A	S19004C	BV605	Biolegend
NKG2D	1D11	BV650	Biolegend
NKp80	5D12	PE	Biolegend
